# Disparities in Liver Transplantation Among Native Americans and Hispanic Individuals: Insights from a Southwest Region Center in the United States of America

**DOI:** 10.3390/jcm15030953

**Published:** 2026-01-24

**Authors:** Anandalakshmi Ponnaluri, Pooja Rangan, Pojsakorn Danpanichkul, Andrew Bell, Rebecca Postagate, Moises Ilan Nevah Rubin, Michael B. Fallon, Karn Wijarnpreecha

**Affiliations:** 1 Department of Internal Medicine, University of Arizona College of Medicine-Phoenix, Phoenix, AZ 85004, USA; 2 Department of Medicine, University of Arizona College of Medicine-Phoenix, Phoenix, AZ 85004, USA; 3 Division of Gastroenterology and Hepatology, Banner University Medical Centre, Phoenix, AZ 85006, USA; 4 Department of Internal Medicine, Texas Tech University Health Sciences Center, Lubbock, TX 79430, USA; 5 Division of Gastroenterology and Hepatology, Department of Internal Medicine, Banner University Medical Centre, Phoenix, AZ 85006, USA; 6 Division of Gastroenterology and Hepatology, Department of Medicine, University of Arizona College of Medicine-Phoenix, Phoenix, AZ 85004, USA; 7 BIO5 Institute, University of Arizona College of Medicine-Phoenix, Phoenix, AZ 85004, USA

**Keywords:** racial and ethnic disparities, barriers at each stage of transplantation, demographic trends, psycho-social issues, post-transplantation outcome

## Abstract

**Background:** Liver transplantation (LT) is the definitive treatment for end-stage liver disease, yet racial and ethnic disparities persist across the LT continuum. This study investigated the patient-level and system-level barriers to LT and evaluated racial disparities in access and outcomes. **Methods:** We conducted a retrospective cohort study (2012–2022) at Banner University Medical Center, Phoenix, analyzing adult LT-referred, evaluated, waitlisted, and transplanted patients. Primary outcomes included mortality and LT barriers, assessed using competing-risk and Cox regression analyses. **Results:** Among 2877 LT-referred patients, 61% were Non-Hispanic White (NHW), 26% Hispanic, 8.8% Native American/Alaska Native (NA/AN), 3% Black, and 1% Asian. Compared with NHW patients, lower rates of LT evaluation and listing were observed among NA/AN (39% and 53%) and Hispanic patients (56% and 63%) versus NHW patients (51% and 64%). Patient-level financial barriers were more common among NA/AN (15.5%) and Hispanic (19.81%) individuals. Waitlist mortality was significantly higher for NA/AN (sub-distribution hazard ratio [SHR]: 5.26; *p* < 0.01) and Hispanic (SHR: 2.92, *p* < 0.02) patients than for NHW patients, whereas graft survival did not differ significantly by race. **Conclusions:** Marked racial and ethnic disparities exist in LT access and waitlist mortality, particularly among NA/AN and Hispanic patients. Targeted interventions addressing financial and systemic barriers are critical to ensuring equitable LT access and improving transplant outcomes.

## 1. Introduction

Liver transplantation (LT) remains the definitive treatment for patients with end-stage liver disease, offering the best chance for survival [1]. Notably, there has been a shift in the demographic composition of transplant recipients, with an increasing proportion of Hispanic and Native American/Alaska Native (NA/AN) individuals undergoing LT. Recent trends show a slight decrease in the number of individuals waitlisted for LT. In contrast, the number of recipients has increased significantly, highlighting ongoing advancements in LT [2]. Cirrhosis, the ninth-leading cause of death in the United States (U.S.), is a major contributor to the rising demand for LT [3]. Additionally, the increasing prevalence of alcohol-associated liver disease (ALD) and metabolic dysfunction-associated steatohepatitis (MASH) among LT recipients reflects evolving patterns in liver disease etiology [4]. Despite the advancements, significant racial and ethnic disparities persist throughout the LT continuum—ranging from assessment to post-LT survival [5,6].

As the U.S. population continues to diversify, racial and ethnic minorities are expected to be the majority by 2050; addressing their healthcare challenges is essential [7]. NA/AN (1.8% of the U.S. population) and Hispanic individuals face high rates of liver disease, including cirrhosis and hepatocellular carcinoma (HCC), with Hispanic individuals further impacted by alcohol use, metabolic disorders [8], and genetic polymorphisms in PNPLA3 [9]. These groups often face barriers to timely diagnosis, treatment, and transplantation, further perpetuating health disparities [10,11,12]. These disparities are particularly pronounced in certain regions, such as the southwestern U.S., where racial and ethnic compositions diverge from national averages. Despite reforms in the LT allocation system, inequities remain in access, and outcomes remain [13].

This study aimed to identify and explore racial and ethnic disparities in LT evaluation, waitlisting, and transplantation processes while proposing strategies to mitigate these inequities.

## 2. Materials and Methods

### 2.1. Study Population and Study Outcomes

This retrospective cohort study included patients referred to Banner University Medical Center Phoenix (BUMCP),Arizona, a large LT referral tertiary care center serving a large and demographically diverse catchment area in the southwestern region of the US. The study population reflects the general regional population, as BUMCP operates as a high-volume transplant center, adhering to national allocation policies set by United Network for Organ Sharing (UNOS) and Organ Procurement and Transplantation Network (OPTN), similarly to other centers in the region. Given its diverse patient base, BUMCP provides a representative sample for analyzing LT outcomes. This study examined barriers to LT and post-transplant outcomes using data from 1 January 2012 to 31 December 2022. Ethical approval was obtained from the Institutional Review Board of BUMCP.

Patients who underwent LT were identified using ICD code 10 related to LT status (ICD-10 code Z94.4). Post-transplant complications were classified under ICD-10 codes T86.40–T86.49. Those waitlisted for LT were identified with ICD-10 code Z76.82. Among the underlying liver diseases leading to transplantation, ALD was the most common indication, classified using ICD-10 codes K70.0, K70.10, K70.11, K70.30–K70.31, and K70.9. MASH, another leading cause, was identified using ICD-10 code K75.81. HCC, a significant primary disease among LT recipients, was classified under ICD-10 code C22.0. Additionally, liver cirrhosis, a common underlying condition among LT candidates, was identified using ICD-10 code K74.60. This study excluded those who did not consider LT candidates at the institution, including those aged ≤18 and ≥80 years and those with uncontrolled sepsis, severe cardiopulmonary disease, multisystem organ failure, and active psychiatric disorders. Patients with missing, unspecified, or multiracial race [566 (16%)] were excluded to reduce race misclassification and analytic heterogeneity. Multivariable analyses used a complete-case approach; missing data were not imputed due to substantial and non-random missingness in key social and clinical covariates. Additionally, manual chart review was conducted for 1438 patients (50%) to validate demographic characteristics and capture detailed pre-LT clinical, barrier-related, and post-LT outcome data not reliably available in structured electronic health record fields; reviewed and non-reviewed patients did not differ meaningfully, indicating minimal risk of systematic information bias.

Then, we performed a longitudinal analysis to determine the barriers to LT, mortality, graft survival, and other clinical outcomes in this cohort. Follow-up time was defined as the last clinical encounter date, death, or study end date (31 December 2022), whichever was the earliest. Barriers were categorized as patient-level (e.g., financial constraints, insurance status, social support) and system-level (e.g., evaluation delays) at all stages of LT, from the referral period to evaluation and transplantation. The primary outcomes were the barriers among candidates undergoing LT evaluation, listing, proceeding with LT, and outcomes after LT.

### 2.2. Statistical Analysis

Continuous variables are summarized as medians and interquartile ranges, and frequencies and percentages are used for categorical variables. Comparative analysis of continuous variables was based on a 2-sample Wilcoxon rank test for samples that failed the Shapiro–Wilk normality test; otherwise, it was based on a 2-sample *t*-test. Comparative analysis of categorical variables was based on a 2-sided chi-square test or Fisher’s exact test, as appropriate. Non-Hispanic White (NHW) patients were used as the comparison group for all comparative analyses. A competing-risk regression analysis was used to model and estimate differences in the cumulative incidence of waitlist mortality (death or being too sick for transplant) between racial groups, with transplant being treated as a competing event.

Analyses were adjusted for confounders and clinically important factors, including age, sex, waitlisted physiological MELD (Model for End-Stage Liver Disease) score, body mass index (BMI) at listing, diabetes mellitus (DM), and employment status. Employment status was included as a proxy measure of individual-level socioeconomic stability and access to healthcare, consistently with prior research on LT disparities demonstrating that individual- and neighborhood-level socioeconomic status are independently associated with transplant outcomes [14]. Parameter estimates are provided as sub-distribution hazard ratios (SHRs), which measure the effects of race and ethnicity on the cumulative incidence of waitlist mortality. According to the Organ Procurement Transplant Network, graft survival was defined as the absence of either recipient death or re-transplantation. The 1-year LT graft survival was estimated using Kaplan–Meier analyses, and comparisons were made between racial groups using the log-rank test. We then performed multivariable Cox proportional hazard analysis using variables selected a priori, which were associated with graft failure (i.e., donor variables—DCD (Donation after Circulatory Death) and cold ischemia time—and recipient variables—age, sex, DM, BMI, MELD score, length of hospital stay, and employment status). Model violations and assumptions, including multicollinearity and proportional hazards, were assessed. Statistical analyses were completed using Stata version 18.0 (College Station, AZ, USA), and *p* values < 0.05 were considered statistically significant.

### 2.3. Ethical Approval

Ethical approval for this single-center study was granted by the Institutional Review Board (IRB). All necessary consent was obtained from the participants, adhering to the institutional protocols. The data for this study were sourced from the BUMCP database accessed after 26 September 2023 after IRB approval and analyzed according to ethical guidelines.

## 3. Results

### 3.1. Demographic Trends of LT Evaluations and Recipients

**Figure 1:**
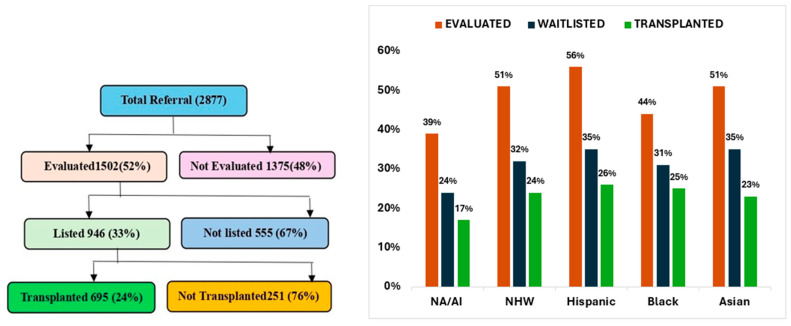
Left. Overview of patient progression from referral to transplantation, showing percentage of patients in each stage. Right. Racial distribution of patient progression from referral to transplantation in percentages. Abbreviations: NA/AN, Native American/Alaska Native; NHW, Non-Hispanic White.

Over 10 years, there were 2877 patients referred for LT evaluation; among these, only 695 (24%) underwent LT (Figure 1); 61% were NHW, 26% Hispanic, 9% NA/AN, 3% Black, and 1% Asian patients.

Key demographic and clinical differences were observed across racial and ethnic groups. NA/AN and Hispanic recipients had higher median BMIs (30 kg/m^2^) compared with other groups. English proficiency varied, with NHW patients the most often proficient (98%) and Hispanic patients the least often proficient (82%). Females represented 55% of NA/AN recipients, contrasting with male predominance in all other groups. NA/AN patients also constituted the majority of those who were divorced or single (52%). Insurance coverage differed by group, with commercial insurance the most common overall and particularly prevalent among NA/AN recipients (62%), whereas Asian individuals had the lowest proportion of commercial coverage (35%). Medicaid, Medicare, self-pay, and VA/other government insurance were used less frequently (Table 1).

### 3.2. Primary Disease Trends

**Figure 2:**
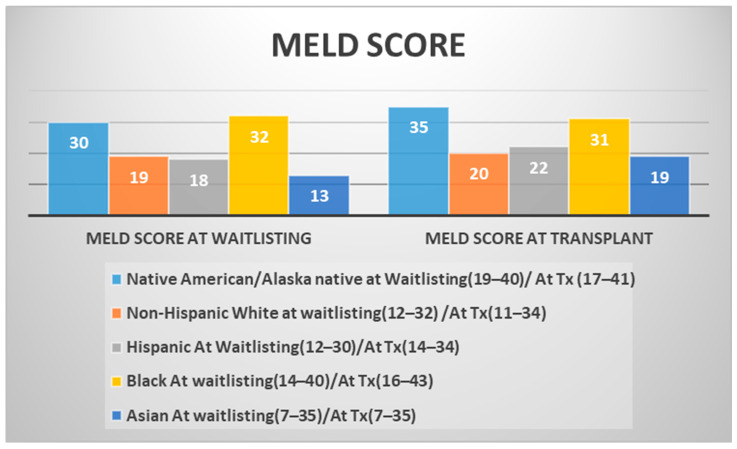
Median MELD scores by race at waitlisting and transplantation. Median Model for End-Stage Liver Disease (MELD) scores at the time of waitlisting and at LT stratified by race/ethnicity. Bars represent unadjusted median MELD scores, with interquartile ranges (IQRs) indicated in parentheses in the legend. This figure presents descriptive, unadjusted comparisons; no multivariable adjustment is applied. Racial and ethnic groups include Non-Hispanic White (NHW), Hispanic, Native American/Alaska Native (NA/AN), Black, and Asian patients.

ALD was the most common cause of liver disease in LT recipients, with the highest prevalence in NA/AN (41%) and NHW (33%) individuals. MASH was another common liver disease, with Hispanic patients showing a slightly higher prevalence (20%) than other groups. HCC was a significant primary disease among LT recipients, particularly among Asian (40%), Hispanic (25%), and Black (26%) individuals (Table 2). At waitlisting, Black patients had the highest median MELD scores (32; IQR: 14–40), followed by NA/AN patients (30; IQR: 19–40). This pattern persisted at the time of LT, with NA/AN recipients exhibiting the highest median MELD score (35; IQR: 17–41), followed by Black recipients (31; IQR: 16–43) (Figure 2).

### 3.3. Barriers at the Time of Evaluation

**Figure 3:**
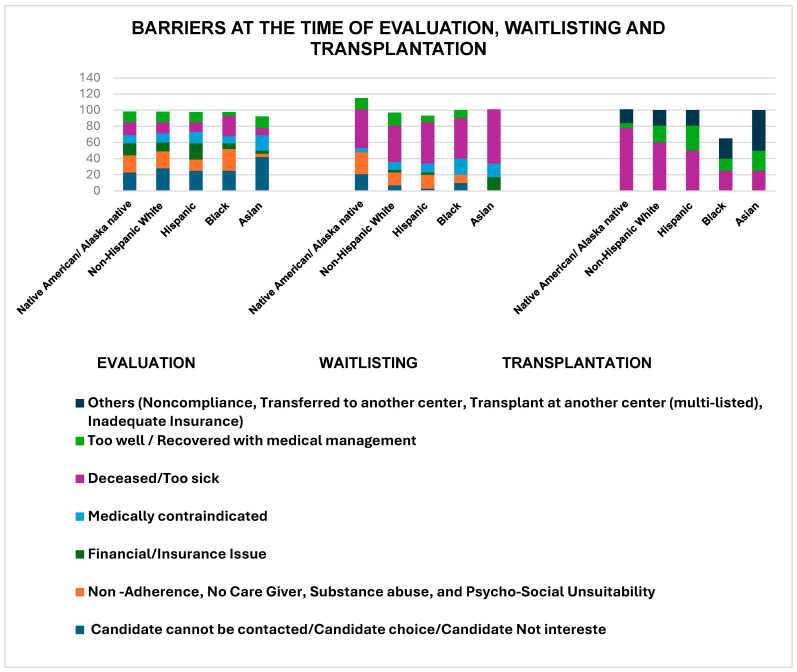
Barriers at the time of evaluation, waitlisting, and transplantation. Bars represent the unadjusted distribution of primary reasons for non-progression at each stage of the LT continuum (evaluation, waitlisting, and transplantation), stratified by race and ethnicity.

**Evaluated versus Not Evaluated:** Among the referred candidates, only 52% were evaluated (Figure 3). The main reasons for not being evaluated included inability to reach the patient or family for a follow-up/the patient no longer being interested in LT (27%), as well as psychosocial unsuitability, non-adherence, lack of a caregiver, and substance abuse (18.98%). Financial and insurance issues were reported as barriers by 13.31% of the candidates. This barrier affected NA/AN (15.5%) and Hispanic patients (19.81%) more significantly than other racial and ethnic groups did (Table 3).

### 3.4. Barriers After Evaluation Until Listing for LT

Among the 1502 (52%) evaluated candidates, 946 (63% of the evaluated) were listed for LT. The percentage of listed candidates varied slightly across racial and ethnic groups (Table 2). The main reasons for not being listed included psychosocial unsuitability, non-adherence, lack of a caregiver, and substance abuse (17%), as well as inability to reach the patient or family for a follow-up/the patient no longer being interested in LT (7%). Financial and insurance issues were reported as barriers by 3% of the candidates. This barrier seems to affect Asian individuals (17%) more significantly than other racial and ethnic groups (Figure 3).

### 3.5. Waitlist Barriers

Among the 946 (33%) candidates listed for LT, 695 (73%) underwent LT. The percentage of transplant candidates varied slightly across racial and ethnic groups, with NHW individuals having the highest percentage (74%) (Table 2). Some candidates (23%) did not undergo transplantation because they were deemed too well or recovered with medical management, particularly among Hispanics individuals (31%) (Figure 3). The median listing time varied across racial and ethnic groups, with Asian individuals having the longest median listing time (199 days) and Black individuals the shortest (16 days).

### 3.6. Referral, Evaluation, Waitlist Mortality

At the time of referral, 196 out of 1427 NA/AN (14%), 22 out of 137 NHW (16%), 123 out of 867 Hispanic (14%), 38 out of 314 Black (12%), and 11 out of 44 Asian (25%) individuals died. Upon evaluation, 263 out of 561 NA/AN (47%), 27 out of 56 NHW (48%), 147 out of 327 Hispanic (45%), 80 out of 156 Black (51%), and 5 out of 10 Asian (50%) individuals were deemed too sick for transplantation.

### 3.7. Waitlist Mortality and Graft Survival

**Figure 4:**
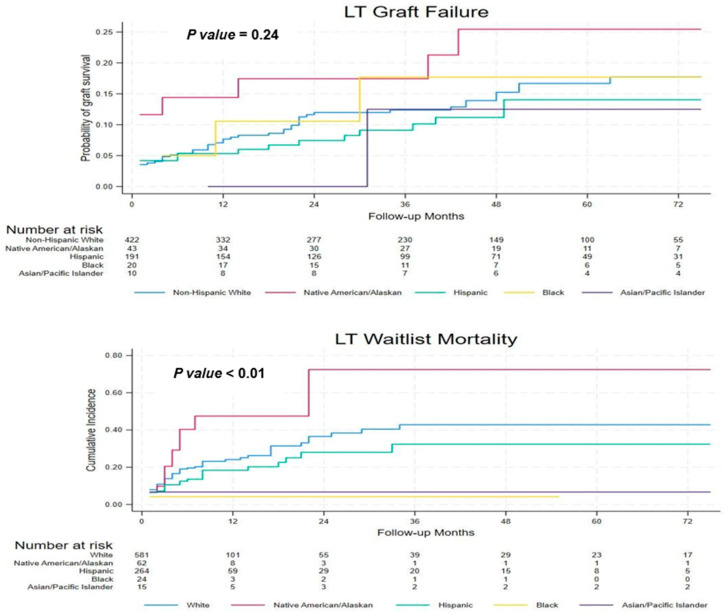
Top Cumulative incidence of graft survival post-liver transplantation. Bottom Cumulative incidence of mortality while on the liver transplant waitlist.Top Kaplan–Meier curves for graft survival post-transplantation. No statistically significant differences in graft failure were observed between racial/ethnic groups (*p* = 0.24). Bottom Cumulative incidence of mortality while on the LT waitlist. Significant differences were observed among racial/ethnic groups, with Native American/Alaska Native (NA/AN) patients exhibiting the highest waitlist mortality (*p* < 0.01). Analyses are unadjusted.

Multivariate competing-risk analysis showed a significantly higher risk of waitlist death for NA/AN and Hispanic patients than for NHW patients (Figure 4). There were no statistically significant differences among the races in terms of post-LT graft survival, as shown in (Supplementary Table S1).

### 3.8. Other Post-LT Complications

We analyzed 695 LT recipients, comprising 427 NHW, 43 NA/AN, 195 Hispanic, 20 Black, and 10 Asian individuals, and analyzed several post-LT complications (Table 4). Within one year post-transplantation, biliary leak occurred in 10% of cases overall, with rates of 12% among NA/AN, 7% among Hispanic, and 20% among Black recipients. Biliary stricture occurred in 11% of cases, with 16% among NHW and 10% among Hispanic individuals, and data were unavailable for other groups. Hepatic artery thrombosis (HAT) occurred in 2% of cases, with 5% in NA/AN and 2% in Hispanic individuals. Hepatic artery stenosis (HAS) affected 13% of the overall population, with 19% among NHW and 12% among Hispanic recipients and higher rates among Black and Asian recipients. Other complications, such as sepsis, hemorrhage, acute renal failure, pancreatitis, cardiac arrest, cerebral vascular incidents, and malignancy, collectively occurred in 16% of the cases, but there were no significant racial disparities.

## 4. Discussion

In this cohort of 2877 LT-referred patients at a large LT referral center in the southwest region of the US from 2012 to 2021, significant disparities were observed compared with NHW patients. Our study examined the demographic, clinical, and social determinants of access to LT across racial and ethnic groups, evaluated disparities across the evaluation, listing, and post-transplantation phases, and underscored the need for targeted interventions to reduce inequality in LT care. Patient- and system-level barriers contribute to disparities at each stage of care, with bias influencing referral and listing processes.

Our retrospective study on LT disparities among NA/AN and Hispanic populations is particularly relevant given Arizona’s demographics, where the state ranks seventh for NA/AN and fourth for Hispanic/Latino populations [15]. The southwest location and rural–urban divide create notable barriers to transplant access. Despite the single-center design, our findings align with OPTN/UNOS and SRTR data demonstrating persistent racial, ethnic, and socioeconomic disparities across regions, including the southwest. Hispanic and Native American candidates face higher barriers related to insurance, socioeconomic disadvantages, and geographic access, which coincide with delayed evaluation and reduced waitlisting [16,17]. These patterns mirror the patient- and system-level barriers observed in our cohort, supporting the generalizability of our findings.

Over the last 10 years, the demographic composition of LT candidates and recipients has revealed notable variations across racial and ethnic groups. The majority of LT recipients were NHW (61% compared to 70.2% in 2011 [18]), Hispanic (26% from 16.9% in 2011 [18]), and NA/AN (9%), making up a growing proportion of recipients. This shift aligns with national demographic trends, in which minority populations are projected to become the majority by 2050 [7]. Differences in clinical and social characteristics were observed across racial and ethnic groups, including higher median BMIs among NA/AN and Hispanic recipients, a factor previously associated with adverse transplant-related outcomes [19,20].

Our multivariate analysis revealed that multiple independent variables were associated with inequalities in LT. ALD and MASH were the most common etiologies across racial and ethnic groups, with ALD more prevalent among NA/AN and NHW recipients and MASH more common among Hispanic recipients. These patterns are consistent with previously reported population-level trends in alcohol-related and metabolic liver disease, including higher burdens of obesity and DM among Hispanic populations [21,22,23,24].

The higher median MELD scores at evaluation among the Black (32) and NA/AN (30) patients indicate more advanced liver disease at presentation, which may reflect different etiologies of liver disease for LT referral (higher HCC than others) and patterns of liver disease progression [25,26]. Elevated MELD scores at LT among NA/AN and Black recipients may reflect later transplantation or greater disease severity at the time of LT, a pattern that has been associated with increased waitlist mortality in prior studies [27].

Similarly, the evaluation process presents barriers to some groups. A major finding of this study was that only 52% of the referred candidates were evaluated, of which 33% were ultimately listed, and 24% underwent LT. Psychosocial factors and non-adherence were common barriers, particularly among NA/AN candidates, while financial challenges were more frequently reported among Hispanic and NA/AN patients. These patterns are consistent with prior studies demonstrating associations between psychosocial barriers, socioeconomic disadvantages, and reduced access to transplantation, including lower listing rates among Hispanic patients even after adjustment for socioeconomic factors [17,28,29,30]. Collectively, these findings highlight the need for targeted strategies such as improved financial support, insurance access, and culturally responsive mental health and addiction services to reduce barriers across the transplant continuum [31,32].

We observed higher waitlist mortality among NA/AN and Hispanic patients compared with NHW patients, consistently with prior reports of racial and ethnic disparities in transplant access and timing [33,34]. These differences may reflect a combination of more advanced disease at listing and social and structural factors influencing access to care [35]. Despite these disparities, our study found no statistically significant difference in graft survival post-transplantation across racial and ethnic groups, suggesting comparable outcomes once transplantation is achieved.

Post-transplantation complications showed some racial differences in the rates of biliary leak, HAT, and HAS. However, the low incidence in each group limits firm conclusions about racial disparities. The higher rates of biliary complications in NA/AN and Black populations require further investigation of potential factors, such as genetics, healthcare access, and post-transplant care. Although sepsis, hemorrhage, and other complications did not differ significantly by race, the overall incidence was 16%, underscoring the need for improved monitoring and management, particularly in high-risk populations.

However, this retrospective, single-center study has several limitations. Selection bias may have occurred due to non-random sampling, and the findings from a single geographic region (Arizona) may not be generalizable to other populations. Exclusion of patients with unspecified or multiracial race (16%) may have introduced selection bias; however, substantial missing data in key social and clinical variables precluded reliable multivariable analysis in this subgroup. The study period spanned multiple years, during which changes in clinical practice, transplantation protocols, or healthcare policies may have occurred, potentially affecting or confounding the outcomes of interest. Key social determinants of health, such as distance to the transplant center, educational attainment, income, and urban versus rural residence, were unavailable, and employment status was used as a proxy, which may not have fully captured socioeconomic disadvantages. Additionally, potential misclassification, unmeasured confounding, and clinician bias during evaluation could not be fully addressed. However, granular data from tertiary-care LT centers with highly diverse patient populations, including NA/AN and Hispanic individuals, are required. These data are crucial because most previous studies had no granular data among diverse racial groups regarding barriers to access to LT evaluations and outcomes, which is a knowledge gap in the field.

## 5. Conclusions

This study underscores the persistent racial and ethnic disparities in the LT process from evaluation and listing to waitlist mortality and post-transplant outcomes. The data point to significant barriers related to financial access, psychosocial factors, and disease severity, particularly among NA/AN and Hispanic populations. Enhanced cultural competence in healthcare delivery, expanded insurance coverage, financial assistance programs, and targeted outreach are needed in underserved communities to ensure equitable access to LT and to improve outcomes for all patients, regardless of their racial or ethnic background.

## Figures and Tables

**Table 1:** Baseline demographic characteristics of LT-referred patients at Banner University Medical Center, Phoenix, between 2012 and 2022.

	Study Population (n = 2877)	Non-Hispanic White (n = 1760)	Native American/Alaska Native (n = 253)	Hispanic (n = 744)	Black (n = 77)	Asian (n = 43)
Age (Y)	58 (50–64)	58 (51–64)	53 (44–60) *	58 (51–63)	57 (41–62)	60 (54–64)
Male	1634 (57%)	1011 (57%)	113 (45%) *	438 (59%)	45 (58%)	27 (63%)
Female	1243 (43%)	749 (42%)	140 (55%) *	306 (41%)	32 (41%)	16 (37%)
BMI (kg/m^2^)	28 (24–32)	28 (24–31)	30 (26–35) *	30 (25–33) *	28 (24–31)	27 (23–30)
**Language**						
English	2710 (94%)	1735 (98%)	252 (99%)	611 (82%) *	77 (100%)	35 (81%) *
Others	167 (6%)	25 (2%)	1 (1%)	133 (18%)	0 (0%)	8 (19%)
**Marital status**						
Divorce/ single/widow	1520 (53%)	941 (53%)	131 (52%)	380 (51%)	39 (50%)	29 (67%) *
Married/life partner	1293 (45%)	781 (44%)	117 (46%)	352 (47%)	31 (40%) *	12 (28%) *
Unknown	64 (2%)	38 (2%)	5 (2%)	12 (2%)	7 (10%)	2 (5%)
**Insurance**						
Commercial	1507 (52%)	910 (52%)	156 (62%) *	399 (54%)	27 (35%) *	15 (35%) *
Medicaid	444 (15%)	290 (16%)	21 (8%) *	109 (15%)	19 (25%)	5 (12%)
Medicare	321 (11%)	177 (10%)	20 (8%)	108 (14%) *	6 (8%)	10 (23%)
Self-pay	456 (16%)	298 (17%)	44 (17%)	90 (12%) *	15 (20%)	9 (20%)
VA/other government	109 (3%)	66 (3%)	7 (3%)	30 (4%)	4 (5%)	2 (5%)
Unknown or missing	40 (1%)	19 (1%)	5 (2%)	8 (1%)	6 (7%)	2 (4%)

Values are presented as n (%) or median (interquartile range). * *p* ≤ 0.05 (all other *p* values > 0.05). Non-Hispanic White (NHW) race was compared with all other groups through pairwise comparisons of *p* values from the chi-squared test for categorical variables and a two-sample Wilcoxon rank test for continuous variables. BMI (body mass index), VA (Veteran Affairs life insurance), and commercial (Blue Cross Insurance, Aetna, and other commercial insurance).

**Table 2:** Primary disease.

	Study Population (n = 2877)	Non-Hispanic White (n = 1760)	Native American/Alaska Native (n = 253)	Hispanic (n = 744)	Black (n = 77)	Asian (n = 43)
Alcoholic liver disease	893 (31%)	574 (33%)	104 (41%) *	201 (27%) *	10 (13%) *	4 (9%) *
Cirrhosis—MASH	403 (14%)	206 (12%)	43 (17%) *	147 (20%) *	4 (5%)	3 (7%)
HCC	616 (21%)	348 (20%)	34 (14%) *	187 (25%) *	20 (26%)	17 (40%)
MELD score at waitlisting	20 (12–32)	19 (12–32)	30 (19–40) *	18 (12–30)	32 (14–40) *	13 (7–35)
Cirrhosis—autoimmune and PBC	126 (4%)	125 (7%)	12 (5%)	36 (5%)	2 (3%)	1 (2%)
Graft failure/re-transplantation	44 (2%)	29 (2%)	7 (3%)	7 (1%)	1 (1%)	0 (0%)
Cirrhosis—cryptogenic	66 (2%)	33 (2%)	5 (2%)	21 (3%)	6 (8%)	1 (2%)
Cirrhosis—drugs and infections	75 (3%)	49 (3%)	4 (2%)	8 (1%)	7 (10%) *	7 (16%)

Values are n (%) or median (interquartile range). * *p* ≤ 0.05 (all other *p* values > 0.05). HCC (hepatocellular carcinoma),), MELD (Model for End-Stage Liver Disease), cirrhosis—MASH (metabolic dysfunction-associated steatohepatitis), and PBC (primary biliary cholangitis).

**Table 3:** Racial disparities in barriers to transplantation at evaluation, waitlisting, and transplantation stages.

BARRIERS AT THE TIME OF EVALUATION					
Total Referred	NA/AN (n = 253)	NHW (n = 1760)	Hispanic (n = 744)	Black (n = 77)	Asian (n = 43)
Patient or family could not be reached for a follow-up/patient was no longer interested in LT	31 (23%)	243 (28%)	83 (25%)	11 (25%)	9 (42%)
Psycho-social unsuitability, non-adherence, no care giver, and substance abuse	29 (21%)	171 (21%)	48 (14%) *	12 (27%)	1 (4%) *
Financial/insurance issue	21 (15%)	94 (11%)	64 (20%) *	3 (7%)	1 (4%)
Medically contraindicated	13 (10%)	96 (11%)	45 (14%)	4 (9%)	4 (19%)
Patient deceased	22 (16%)	123 (14%)	38 (12%)	11 (25%) *	2 (9%)
Too well/recovered with medication	18 (13%)	112 (13%)	41 (13%)	2 (5%)	3 (14%)
**BARRIERS AT THE TIME OF WAITLIST**					
Patient or family could not be reached for a follow-up/patient was no longer interested in LT	12 (21%)	24 (7%)	5 (3%)	1 (10%)	0 (0%)
Psycho-social unsuitability, non-adherence, no care giver, and substance abuse	15 (27%) *	52 (16%)	27 (17%)	1 (10%)	0 (0%)
Financial/insurance issue	0 (0%)	11 (3%)	5 (3%)	0 (0%)	1 (17%)
Medically contraindicated	3 (5%)	32 (10%)	17 (11%)	2 (20%) *	1 (17%)
Deceased/too sick	27 (48%)	147 (45%)	80 (51%) *	5 (50%)	4 (67%)
Too well	8 (14%)	53 (16%)	12 (8%)	1 (10%)	0 (0%)
**BARRIERS AT THE TIME OF TRANSPLANTATION**					
Died/too sick	14 (78%) *	91 (60%)	34 (50%)	1 (25%)	1 (25%)
Too well/recovered with medical management	1 (6%)	32 (21%)	21 (31%)	2 (15%)	1 (25%)
Others	3 (17%)	29 (19%)	13 (19%)	1 (25%)	2 (50%)

Abbreviations: LT, liver transplantation; NA/AN, Native American/Alaska Native; NHW, Non-Hispanic White. * *p* ≤ 0.05 (all other *p* values > 0.05).

**Table 4:** Complications within 1-year post-transplantation.

Transplanted (695)	NHW (427)	NA/AN (43)	Hispanic (195)	Black (20)	Asian (10)
**Biliary Leak Within 1 Year Post-Tx**	42 (10%)	5 (12%)	14 (7%)	4 (20%)	0 (0%)
**Biliary Stricture Within 1 Year Post-Tx**	49 (11%)	7 (16%)	20 (10%)	0 (0%)	0 (0%)
**HAT Within 1 Year Post-Tx**	7 (2%)	2 (5%)	3 (2%)	0 (0%)	0 (0%)
**HAS Within 1 Year Post-Tx**	55 (13%)	8 (19%)	23 (12%)	4 (20%)	2 (20%)
**Other Complications Within 1 Year Post-Tx (sepsis, hemorrhage, acute renal failure, pancreatitis, cardiac arrest, cerebral vascular, malignancy)**	67 (16%)	8 (19%)	10 (5%)	8 (40%)	2 (20%)

Abbreviations: NA/AN, Native American/Alaska Native; NHW, Non-Hispanic White; HAT, Hepatic Artery Thrombosis; HAS, Hepatic Artery Stenosis.

## Data Availability

Data are available on request due to privacy/ethical restrictions.

## Supplementary Materials

The following supporting information can be downloaded at https://www.mdpi.com/article/10.3390/jcm15030953/s1. **Table S1:** Waitlist Mortality and Graft Survival among Liver Transplant recipients at Banner University Medical Center Phoenix, between 2012–2022.

## Abbreviations

The following abbreviations are used in this manuscript:

AHR	Adjusted Hazard Ratio
ALD	Alcohol-Associated Liver Disease
BUMCP	Banner University Medical Center, Phoenix
BMI	Body Mass Index
CI	Confidence Interval
DM	Diabetes Mellitus
HAS	Hepatic Artery Stenosis
HAT	Hepatic Artery Thrombosis
HCC	Hepatocellular Carcinoma
IQR	Interquartile Range
LT	Liver Transplantation
MASH	Metabolic Dysfunction-Associated Steatohepatitis
MELD	Model for End-Stage Liver Disease
NA/AN	Native American/Alaskan Natives
NASH	Nonalcoholic Steatohepatitis
NHW	Non-Hispanic White
OPTN	Organ Procurement and Transplantation Network
PBC	Primary Biliary Cholangitis
SHR	Sub-Distribution Hazard Ratios
SRTR	Scientific Registry of Transplant Recipients
U.S.	United States
UNOS	United Network for Organ Sharing
VA	Veteran Affairs
