# Supplementary material for: Disparities in Liver Transplantation Among Native Americans and Hispanic Individuals: Insights from a Southwest Region Center in the United States of America
Source: J Clin Med. 2026 Jan 24;15(3):953. doi: 10.3390/jcm15030953 (PMC12898143; doi:10.3390/jcm15030953)
Supplement: Supplementary file 1 [file jcm-15-00953-s001.zip › jcm-4091885-supplementary.pdf]

Journal of Clinical Medicine

Supplementary Table S1. Waitlist Mortality and Graft Survival among Liver Transplant recipients at Banner University Medical Center Phoenix between 2012-2022.

	**Waitlist Mortality**		**Graft Survival**	
	**SHR (95% CI)**	***P*- value**	**SHR (95% CI)**	***P*- Value**
**Native American / Alaskan Natives**	5.26 (1.94 – 14.22)	< 0.01*	2.25 (0.66 – 7.61)	0.19
**Hispanic**	2.92 (1.21 – 7.03)	< 0.02*	1.17 (0.42 – 3.22)	0.76
**Black**	0.80 (0.09 – 6.61)	0.84	1.60 (0.35 – 7.24)	0.54
**Asian and Pacific Islander**	1.33 (0.15 – 11.66)	0.8	1.11 (0.12 – 9.97)	0.92

Abbreviations: CI, confidence interval; SHR, Sub-distribution Hazard Ratio. *Values are significant P <0.05.
